# Molecular Characterization of Feline Chaphamaparvovirus (*Carnivore chaphamaparvovirus 2*) Firstly Detected in Dogs from China

**DOI:** 10.1155/2023/5882871

**Published:** 2023-03-14

**Authors:** Jun Ji, Hao Cui, Shuqi Xu, Xin Xu, Qiang Liu, Yunchao Kan, Qingmei Xie, Lunguang Yao

**Affiliations:** ^1^Henan Provincial Engineering Laboratory of Insects Bio-Reactor, Henan Provincial Engineering and Technology Center of Health Products for Livestock and Poultry, Henan Provincial Engineering and Technology Center of Animal Disease Diagnosis and Integrated Control, Nanyang Normal University, Nanyang 473061, China; ^2^Zhongjing Research and Industrialization Institute of Chinese Medicine, Zhongguancun Scientific Park, Meixi, Nanyang, Henan 473006, China; ^3^College of Animal Science, South China Agricultural University, Guangzhou 510642, China

## Abstract

A new type of parvovirus known as feline chaphamaparvovirus (FeChPV) was discovered in the feces of shelter cats in Canada in 2019, and >50% of cats were reported to be infected with this virus. In this study, two FeChPV-positive samples were identified from the rectal swabs of 285 dogs with diarrhea but none in 50 healthy dogs. Whole genome sequences of these two FeChPV strains (OQ162042 and OQ162043) were amplified and compared with those of the two viruses originally discovered in Canada (IDEXX-1 and VRI849). The whole genome, NS1, and VP1 of the two FeChPV strains shared a high identity of 95.0%–97.8% nucleotide, 96.9%–98.6% amino acid (aa), and 97.2%–98.8% aa with the reported FeChPV strains, respectively. The phylogenetic tree of NS1 and VP1 revealed that two FeChPV strains, namely, CHN20201025 and CHN20201226, were closely clustered with the two FeChPV prototypes detected in Canada in a group. Moreover, CHN20201025 and CHN20201226 were obviously different from *Carnivore chaphamaparvovirus 1* and were classified as C*arnivore chaphamaparvovirus 2*. This is the first study to report the identification of FeChPV in fecal samples from dogs in China, and the genetic analysis of the FeChPV, which was previously detected in Canadian cats, would improve our understanding of its host spectrum.

## 1. Introduction

Parvoviruses (family Parvoviridae) are small, nonenveloped, icosahedral, single-stranded linear DNA viruses measuring 3.9–6.3 kb in size [1]. Historically, the family Parvoviridae is divided into two subfamilies, Densovirinae and Parvovirinae, which include viruses that primarily infect invertebrates and vertebrates, respectively [2]. However, according to an update on Parvoviridae taxonomy by The International Committee on Taxonomy of Viruses (ICTV), the novel Hamaparvovirinae subfamily was introduced that includes the genera *Hepanhamaparvovirus*, *Penstyrhamaparvovirus*, *Brevihamaparvovirus*, *Ichthamaparvovirus*, and *Chaphamaparvovirus* [3].

With the continued discovery of chaphamaparvoviruses (ChPVs) in various types of animals, researchers are giving increased attention to this new form of virus. Metagenomic sequencing of oropharyngeal swab samples obtained from a fruit bat (*Eidolon helvum*) was performed to first identify ChPVs [4]. The name of these viruses is derived from the acronym CHAP, which refers to the host groups (chiropteran, avian, and porcine) in which they were first identified [4–6].

ChPV was first detected in 2017 in the fecal samples of two dogs with hemorrhagic diarrhea of unknown origin in the United States and subjected to next-generation sequencing. To date, several different animal species have been reported to harbor ChPVs, such as dogs, cats, and turkeys [7–9]. In a previous report, the *Carnivore chaphamaparvovirus 1* species was established based on the complete sequence of the nonstructural protein 1 (NS1) of ChPVs, and all dog and cat strains were classified as *Carnivore chaphamaparvovirus 1* species [10]. Subsequently, a novel virus belonging to the same genus was detected in the feces of cats with diarrhea; this novel virus was categorized as *Carnivore chaphamaparvovirus 2* species by the ICTV [11].

In this study, we aimed to investigate FeChPV in fecal samples from dogs in China and genetic analysis of detected viruses.

## 2. Materials and Methods

### 2.1. DNA Extraction and Virus Detection

Rectal swabs were collected from 335 dogs (50 healthy and 285 with diarrhea) in pet hospitals in Henan, Jiangsu, Anhui, and Inner Mongolia from February 2019 to June 2020. For the extraction of nucleic acid, the collected swabs were rinsed in 1 mL of phosphate-buffered saline. The virus DNAs/RNAs were extracted using the EasyPure Viral DNA/RNA Kit (TransGen Biotechnology, Inc., Beijing, China) and stored at −80°C until use.

#### 2.1.1. Virus Screening

Canine coronavirus (CCoV) [12], CachaV [13], canine distemper virus (CDV) [14], canine rotavirus (CRV) [15] , and canine parvovirus (CPV-2) [16] were detected via PCR/RT-PCR as described previously, and FeChPV was detected via nested PCR [11]. All abovementioned viral strains were used to detect diarrhea-related diseases in dogs using rectal swabs. The primers used in previous studies and those designed in the present study are shown in Supplementary Table 1. Nested PCR was performed under the following reaction conditions: 95°C for 5 min, 35 cycles of denaturation at 95°C for 30 s, annealing for 30 s at 49°C for the first round and 51°C for the second round of primers, 72°C for 42 s, and finally 72°C for 10 min of final extension. The infection status of the samples was determined using a Wayne diagram (https://jvenn.toulouse.inra.fr/app/example.html) and visualized using the UpSet map package in the TBtools software [17, 18].

### 2.2. Complete Genome Amplification

Based on the viral sequencing performed in this study, four sets of customized primers (FeChPV-F1/R1, FeChPV-F2/R2, FeChPV-F3/R3, and FeChPV-F4/R4, displayed in Supplementary Table 1) for the whole FeChPV genome were created. A 20 *μ*L reaction mixture consisting of a template DNA (>100 ng/L), 6 pmol of upstream/downstream primers, PrimeSTAR HS DNA polymerase, a supporting reaction buffer was used to amplify genes (TaKaRa Biotechnology Co., Ltd., Dalian, China). The following cycle conditions were applied for sequence amplification: initial denaturation at 95°C for 5 min, followed by 35 cycles of denaturation at 95°C for 30 s, annealing at 55°C `for 30 s, extension at 72°C for 1 min, and then a final extension at 72°C for 10 min. After obtaining the amplicons, they were cloned into a pMD18-T simple vector (TaKaRa Biotechnology Co., Ltd., Dalian, China) for further sequencing (Syn-Biotechnology, Suzhou, China).

### 2.3. Identity and Phylogenetic Analyses

Pairwise sequence alignment was performed using the Clustal W model in the MegAlign program of the Lasergene 7.0 software (DNASTAR Inc., Madison, WI, USA). The sequence alignment Heatmap was implemented and visualized using ChiPlot (https://www.chiplot.online/). A phylogenetic tree was constructed based on the amino acid (aa) sequences of NS1 and VP1 for evolution analysis using the MEGA X software with the LG + G + F model and 1000 bootstrap replicates [19].

### 2.4. Mutation and Protein Structure Prediction for NS1 and VP1

The obtained sequences of the FeChPV strains were compared with those of the reference strains. The altered amino acid sequences were predicted and modeled using SWISS-MODEL (https://swissmodel.expasy.org/interactive) and Phyre2 (https://www.sbg.bio.ic.ac.uk/phyre2/html/page.cgi?id=index) according to various amino acid locations, and PDB files were generated after modeling using PyMOL for collation and preservation [20].

### 2.5. Viral Isolation

The supernatant of the treated FeChPV-positive sample was filtered through a 0.22 *μ*m membrane and injected into *Crandell Rees* feline kidney (CRFK, derived from cats) cells and *Madin Darby* canine kidney (MDCK, derived from dogs) cells under 5% carbon dioxide. The cells were subjected to continuous passage till the fifth generation, and alterations in CRFK and MDCK cells were tracked.

## 3. Results

### 3.1. Positive Rate and Coinfection Status

**Figure 1:**
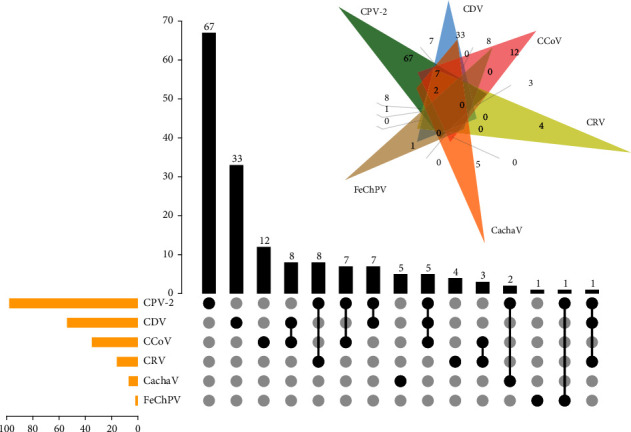
The positive rates of FeChPV, CCoV, CachaV, CRV, CDV, and CPV-2. The coinfection of six pathogens is illustrated via a Venn diagram and an UpSet plot. The UpSet plot presents the distribution of different viruses in the samples. The bar chart above represents the number of genes contained in each type of group. The bar chart at the bottom left represents the number of positive numbers included in each type of pathogen. The dotted line at the bottom right presents the types of events contained in the group.

After screening for viruses, 2 of 285 dogs with diarrhea and 0 of 50 healthy dogs were positive for FeChPV. Dog (CHN20201025) was a 3-month-old male Labrador retriever that was coinfected with CPV-2. In turn, FeChPV was detected alone in dog (CHN20201226), which was a 4-month-old female toy poodle. Both of the two positive dogs were suffered from acute gastro-enteritis without underlying disease. Chi-square analysis revealed a *p*-value of 0.089, which indicated that there was no correlation between the presence of the virus and clinical signs.  Figure 1 shows in detail the infection and positive rates of samples from dogs with diarrhea.

### 3.2. Sequence Identities

The complete genome of CHN20201025 and CHN20201226 was 4092 nucleotides (nt) in length, and all sequences were deposited in GenBank (OQ162042 and OQ162043). The FeChPV genome had two major ORFs, one encoding a 658-aa NS1 and the other encoding a 508-aa capsid protein (VP1). The NS1 protein in the two Chinese strains contained two conserved replication initiation motifs, 95FHIHVMAL102 and 149SLIAYMCK156 [21]. It also contained the highly conserved Walker motifs 311GCSNTGKS318, 349IGVWEE354, 366KQIFEGMECSIPVK379, and 391IIMTTN396 of the helicase domain as reported previously [22].

**Figure 2:**
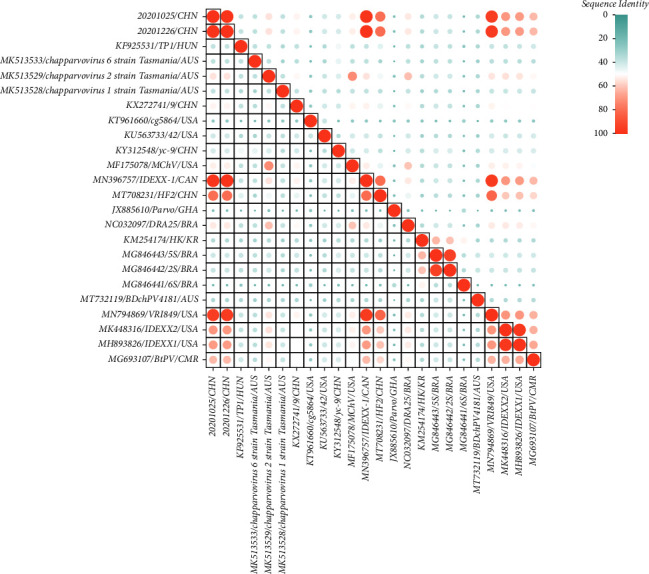
Sequence identity of FeChPV and other strains; on both sides of the main figure are the virus strains and in the middle is the similarity value (upper right: different identity values are expressed in progressive colors ranging from 0% to 100%).

Sequence analyses revealed a 99.2% nt sequence similarity between the two Chinese FeChPV strains, and the strains shared an overall 95.0%–97.8% nt identities of complete sequences, 96.9%–98.6% aa of NS1, and 97.2%–98.8% aa of VP1 with the two prototype FeChPV strains (IDEXX-1, MN396757; VRI849, MN794869). Furthermore, compared with HF2 (accession numbers: MT708231), the two Chinese FeChPV strains in this study had identities of 82.1%–82.8% nt for the complete sequence, 95.2%–96.3% aa identities for NS1, and 97.7%–98.9% aa identities for VP1 (MT708231). Meanwhile, the complete genome of the other member of the genus *Parvovirus* showed a variation of 21.8%–70.6%. The sequence identity of FeChPV and other strains is presented in Figure 2. The identity comparison data are shown in Table 1.

### 3.3. Phylogenetic Tree Analysis for NS1 and VP1

**Figure 3:**
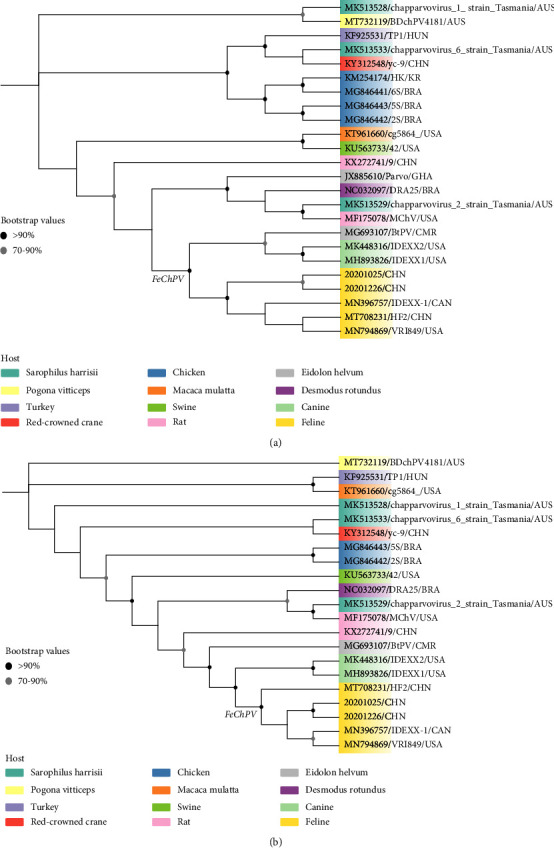
Phylogenetic tree of the NS1 (a) and VP1 (b) genes of FeChPV and other representative reference sequences available in GenBank.

The phylogenetic trees generated for NS1 and VP1 of FeChPVs are shown in Figures 3(a) and 3(b). A thorough examination of these trees revealed that the two Chinese FeChPV strains isolated from dogs were closely related to VRI849, the feline ChPV isolate IDEXX-1, and the Chinese HF2 strain, which belong to the *Carnivore chaphamaparvovirus 2* group, and were clustered into the same branch. The two cachaviruses IDEXX1 and IDEXX2 were distant from the two Chinese FeChPV strains and belonged to another branch.

### 3.4. Mutation and Structural Analysis of NS1 and VP1

**Figure 4:**
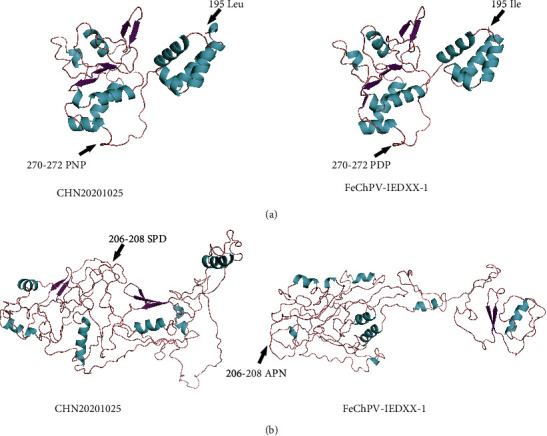
Predicted tertiary structural model of NS1 and VP1 of FeChPV. (a) Tertiary structural model of NS1; (b) tertiary structural model of VP1. “⟵” and letter indicate specific amino acid sequences with specific colors.

A comparison of the amino acid sites of NS1 and VP1 of the two Chinese FeChPV strains and other FeChPV strains revealed the presence of some representative mutation sites in NS1 and VP1 that were shown in Tables 2 and 3. In NS1, there were mutations at Thr79Ala, Ile129Val, Phe414Leu, and Arg512Gly only in these two strains. In VP1, there were mutations at Gly112Arg, Leu74Pro, Sers325Arg, and Thr444Ala, whereas CHN20201025 harbored mutations at only Met19Thr, Phe151Ser, Ala206Ser, and Glu352Gly. We also compared the predicted tertiary structures of NS1 and VP1 of CHN20201025 with those of the FeChPV strain IDEXX-1.  Figure 4 shows the predicted structural changes according to the locations of the mutations.

### 3.5. Virus Cultivation

Until the fifth generation, no specific cytopathic effect (CPE) was observed in MDCK and CRFK cells, and the DNA of FeChPV was not detected using the above-described nested PCR method.

## 4. Discussion

FeChPV was first discovered in the feces of cats in Canada in 2019. Approximately 50% of the cats were infected with this novel ChPV, and clinical history had shown that this virus may be responsible for vomiting and diarrhea in cats [11]. In the present study, considering the current trade and exchange of raising pets at home and abroad, we collected rectal swabs from 50 healthy dogs and 285 dogs with diarrhea and obtained two FeChPV strains only from the dogs with diarrhea (0.7%, 2/285). However, statistical analysis revealed no association between the presence of the virus and the symptoms of diarrhea (*p*  >  0.05), which was compatible with previous report [11]. In particular, one pet dog with enteritis tested positive for FeChPV and not infected with other diseases. According to the clinical history and owner's statement, the two dogs positive for FeChPV were housed without cats but were household with an outdoor access. Herein, infection of FeChPV in the two dogs is whether caused by contacting with cats remains to be determined.

To date, only the mouse kidney parvovirus, belonging to the genus *Chaphamaparvovirus* that causes severe chronic interstitial nephropathy and renal failure, has been isolated from laboratory and field mice populations [23]. In this study, FeChPV has not been isolated from cell lines. Therefore, further research must be conducted using larger, more organized studies or experimental infections to determine whether FeChPV can act as an intestinal pathogen on its own or in concert with other enteroviruses [10].

According to the complete genome sequence analysis, the two newly discovered Chinese FeChPV strains were closer to the Canadian strains (97.2–98.8%) and the reference Chinese HF2 strain (97.7–98.9%) in terms of aa similarity for VP1. Analysis of the phylogenetic tree of NS1 and VP1 showed that the two viruses recovered from Chinese pet dogs and the other three feline strains (two prototype Canadian FeChPV strains and one Chinese FeChPV strain) formed a monophyletic group that should be assigned to *Carnivore chaphamaparvovirus 2*, which were quite distinct from the two cachaviruses that were classified as *Carnivore chaphamaparvovirus 1*.

By comparing the amino acid site mutations between CHN20201025 and IDEXX-1 (prototype Canadian FeChPV strain), we discovered that some mutations in viral proteins altered their tertiary structure model. The structure prediction diagram of NS1, shown in Figure 2, indicated that the predicted tertiary structures of the two FeChPV strains overall showed relatively slight differences from one another, although the mutation sites of Leu195Ile and PNP270-272PDP altered a part of the tertiary structure. In contrast, there were significant differences between the two models of VP1. In addition to the intrinsic model adopted in Phyre2, which utilized different prediction methods from homologous modeling, the other supposed reason for the difference is that there are several mutation sites on VP1, such as Gly112Arg, Phe151Sers, Leu174Pro, and SPD206-208APN. Meanwhile, VP1, the capsid protein, plays a key role in modulating the interaction between parvovirus and its host [10]. A significant number of amino acid site changes may be associated with continuous mutation of the virus to adapt to the stress of antibodies, besides its adaptation to different hosts. In the future, a substantial amount of experimental data should be accumulated to determine whether these mutation sites have an effect on the pathogenicity of the virus.

## 5. Conclusion

In conclusion, we collected data on the identification and genome characterization of FeChPV in Chinese pet dogs for the first time. More in-depth research and accurate virus challenge experiments are required to determine the effect of FeChPV on the health of dogs.

## Figures and Tables

**Table 1:** Sequence identities of the two FeChPV strains identified in this study with those of other ChPV members.

Virus strains	Accession no.	Sequence identity (%)			Genetic distance of VP1
		Genome (nt)	NS1 (aa)	VP1 (aa)	
Turkey parvovirus-TP1	KF925531	39.7–39.8	34.4–34.6	5.31–5.33	0.862
Tasmanian devil-6	MK513533	36.1–36.3	32.5–32.8	22.5–22.7	0.610
Tasmanian devil-2	MK513529	54.1-54.2	47.4–47.7	45.2–45.7	0.359
Tasmanian devil-1	MK513528	39.5–39.7	30.2–30.3	25.9–26.3	0.652
Simian parvo-cg5864	KT961660	27.4-27.5	31.1–31.3	4.92–4.93	0.850
Rat parvovirus 2	KX272741	51.5-51.6	42.0-42.1	43.1–43.7	0.377
Porcine parvovirus 7	KU563733	42.4–42.5	35.3-35.4	34.7–34.9	0.508
Parvoviridae yc-9	KY312548	42.1-42.2	34.0–34.3	32.9–33.2	0.491
Murine chapparvovirus	MF175078	52.2-52.3	44.7–44.9	44.2–44.8	0.371
Feline IDEXX-1	MN396757	95.0–95.9	97.5–98.6	97.2–98.8	0.017
Eidolon helvum-2	JX885610	22.8–22.9	35.5–35.6	NA	NA
*Desmodus rotundus*	NC032097	53.1–53.2	46.2–46.4	46.7–47.3	0.329
Chicken-HK	KM254174	34.5–34.6	29.2–29.5	NA	NA
Chicken-RS/BR/15/5S	MG846443	41.0–41.2	29.5–29.8	28.1–28.4	0.580
Chicken-RS/BR/15/2S	MG846442	39.6–39.8	29.5–29.7	27.2–27.5	0.580
Chicken-RS/BR/15/6S	MG846441	21.8–21.9	28.7–29.0	NA	NA
Chapparvovirus-BDchPV4181	MT732119	34.1–34.2	26.4–26.5	24.5–24.8	0.539
Feline HF2	MT708231	82.1–82.8	95.2–96.3	97.7–98.9	0.017
Carnivore-VRI 849	MN794869	97.0–97.8	96.9–98.0	97.6–98.8	0.017
Cachavirus-1B	MK448316	69.8–69.9	65.1–65.2	65.0–65.1	0.173
Cachavirus-1A	MH893826	70.5–70.6	65.2–65.3	65.1–65.2	0.173
Bat parvovirus	MG693107	64.6–64.7	57.7–57.8	51.2–51.6	0.299

NA: not available.

**Table 2:** Statistics of the main amino acid mutation sites in the NS1 capsid protein of FeChPV in the Chinese strains and reference strains.

Strains	Substitution of amino acid residues in NS1												
	4	2	3	4	7	13	19	23	27	41	44	58	61
		2	4	3	5	7	6	7	8	0	4	2	9
CHN20201025a	S	I	S	I	A	D	L	H	C	L	E	M	T
CHN20201226	S	I	S	I	A	D	I	H	C	L	G	V	T
Feline-HF2-CNC	I	V	S	M	T	H	L	R	G	F	G	V	T
Feline-IDEXX-USA	S	I	F	M	T	D	L	H	C	F	G	V	A
Carnivore-VRI 849-USA	S	V	S	M	T	D	L	H	C	F	G	V	A

**Table 3:** Statistics of the main amino acid mutation sites in the VP1 capsid protein of FeChPV in the Chinese strains and reference strains.

Strains	Substitution of amino acid residues in VP1												
	1	11	15	17	19	20	21	33	35	41	44	49	50
	9	2	1	4	7	6	2	7	2	9	4	3	7
CHN20201025	T	R	F	P	T	A	Q	S	G	N	A	N	E
CHN20201226	M	R	S	P	T	S	H	S	E	H	A	N	D
Feline-HF2-CNC	M	G	F	L	S	S	H	A	E	H	T	N	D
Feline-IDEXX-USA	M	G	F	L	S	S	H	S	E	H	T	N	D
Carnivore-VRI 849-USA	M	G	F	L	S	S	H	S	E	H	T	D	D

## Data Availability

All data generated or analyzed during this study are included in this article. The datasets used and/or analyzed during the current study are available from the corresponding author upon reasonable request.

## Supplementary Materials

Supplementary Materials Table S1. Primers used in this study for the detection of viruses. Supplementary Materials
