# Supplementary material for: Molecular Characterization of Feline Chaphamaparvovirus (Carnivore chaphamaparvovirus 2) Firstly Detected in Dogs from China
Source: Transbound Emerg Dis. 2023 Mar 14;2023:5882871. doi: 10.1155/2023/5882871 (PMC12016959; doi:10.1155/2023/5882871)
Supplement: Supplementary Materials — Table S1. Primers used in this study for the detection of viruses. Supplementary Materials [file 5882871.f1.docx]

Table S1. Primers used in this study for the detection of viruses

| Primer name | Primer sequence (5′–3′) | Target | Location | Reference |
| --- | --- | --- | --- | --- |
| FeChPV-F1 | ATGTGGTTGTGTAGGACA | Sequencing | 36-53^a^ | This study |
| FeChPV-R1 | TCTCTTTCTAAACTGCGTCT |  | 940-959 |  |
| FeChPV-F2 | AACCTCTTCCATACGCAAA |  | 806-824 |  |
| FeChPV-R2 | CGCTGTCATCAATAGTCTCG |  | 1972-1953 |  |
| FeChPV-F3 | CTAATAAGCCCTGAATTAGCC |  | 1675-1695 |  |
| FeChPV-R3 | GCTTATATCAGTTGCCCAT |  | 3097-3115 |  |
| FeChPV-F4 | GAGCACAAGATGATGTCTATGA |  | 2904-2925 |  |
| FeChPV-R4 | TATGGGAGGGGGAATTGAAGTA |  | 4105-4126 |  |
| CCoV-F | CACTAAACTCAAAATGTTGATTC | Detection | 5′UTR | van Nguyen et al., 2017 |
| CCoV-R | TTAAGGATTAAAAACATATTCTA |  | ORF3 |  |
| CachaV-OF | CAACTAGCCGAATGCAGGGA | Detection | NS1 | Fahsbender et al., 2019 |
| CachaV-OR | CGATAACATCCCCGGACTGG |  |  |  |
| CachaV-IF | AGCTCAGTTTGGCCCAGATC |  |  |  |
| CachaV-IR | AGAGGGATCGCTGGATCTGT |  |  |  |
| CDV-F | AGCTAGTTTCATCTTAACTATCAAATT | Detection | N | Wang, et al., 2017 |
| CDV-R | TTAACTCTCCAGAAAACTCATGC |  |  |  |
| CRV-F | GACGGVGCRACTACATGGT | Detection | VP6 | Charoenkul et al., 2021 |
| CRV-R | GTCCAATTCATNCCTGGTGG |  |  |  |
| CPV-2-F | AGAGACAATCTTGCACCAAT | Detection | VP1 | Han et al., 2015 |
| CPV-2-R | ATGTTAATATAATTTTCTAGGTGCT |  |  |  |
| FeChPV-OF1 | GGTGCGACGACGGAAGATAT | Detection | NS1 | Li et al., 2020 |
| FeChPV-OR1 | CAACACCACCATCTCCTGCT |  |  |  |
| FeChPV-OF2 | GCTGCAGTTCAGGTAGCTCA |  |  |  |
| FeChPV-OR2 | CAACACCACCATCTCCTGCT |  |  |  |

**^a^** The primer position is based on the sequence of the VRI-849 strain, GenBank accession number: MN794869.

**REFERENCES**

Charoenkul, K., Janetanakit, T., Bunpapong, N., Boonyapisitsopa, S., Tangwangvivat, R., Suwannakarn, K., ... (2021). Molecular characterization identifies intra-host recombination and zoonotic potential of canine rotavirus among dogs from Thailand. *Transboundary and emerging diseases, 68*, 1240-1252. https://doi.org/10.1111/tbed.13778

Fahsbender, E., Altan, E., Seguin, M.A., Young, P., Estrada, M., Leutenegger, C., & Delwart, E. (2019). Chapparvovirus DNA Found in 4% of Dogs with Diarrhea. *Viruses, 11*, 398. https://doi.org/10.3390/v11050398

Han, S.C., Guo, H.C., Sun, S.Q., Shu, L., Wei, Y.Q., Sun, D.H., ... (2015). Full-length genomic characterizations of two canine parvoviruses prevalent in Northwest China. *Archives of microbiology, 197*, 621-626. https://doi.org/10.1007/s00203-015-1093-4

Li, Y., Gordon, E., Idle, A., Altan, E., Seguin, M.A., Estrada, M., ... (2020). Virome of a Feline Outbreak of Diarrhea and Vomiting Includes Bocaviruses and a Novel Chapparvovirus. *Viruses, 12* . https://doi.org/10.3390/v12050506

van Nguyen, D., Terada, Y., Minami, S., Yonemitsu, K., Nagata, N., LE, T.D., ... (2017). Characterization of canine coronavirus spread among domestic dogs in Vietnam. *The Journal of veterinary medical science, 79*, 343-349. https://doi.org/10.1292/jvms.16-0538

Wang, J., Wang, J., Li, R., Liu, L., & Yuan, W. (2017). Rapid and sensitive detection of canine distemper virus by real-time reverse transcription recombinase polymerase amplification. *BMC veterinary research, 13*, 241. https://doi.org/10.1186/s12917-017-1180-7
